# Bridging Generations: Social Perceptions and Support in Intergenerational Networks

**DOI:** 10.1093/geroni/igaf122.1591

**Published:** 2025-12-31

**Authors:** Claire Growney, Laura Carstensen, Merril Silverstein

## Abstract

As life expectancy increases and family structures evolve, opportunities for intergenerational relationships are expanding, creating new possibilities for social connections, support exchanges, and mutual influence across generations. These relationships can foster social integration, promote well-being, and shape perceptions of aging. Understanding the support behaviors that characterize intergenerational relationships and the perceptions that shape these connections is essential for identifying when and how they provide benefits. This symposium brings together studies examining intergenerational dynamics using diverse methodologies, including family and social network analysis, surveys, and experimental design, to examine implications for older and younger generations. Growney will present findings from the St. Louis Personality and Intergenerational Network study examining associations between socioemotional engagement with grandchildren and well-being outcomes for grandparents. De Fries will explore patterns of social support in older adults using latent class analysis, highlighting how different configurations of intergenerational and non-intergenerational relationships relate to social support and loneliness. Pot will discuss an experimental study about age-based stereotypes, suggesting that representations of older adults as individuals are associated with more positive and less negative stereotyping, which in turn relate to intergenerational affinity. Suitor will examine the intergenerational transmission of parental favoritism and disfavoritism, showing that midlife adults who perceive favoritism from their own parents are more likely to differentiate among their own children in similar ways. The session will conclude with a discussion led by Merril Silverstein, integrating insights from these studies and exploring broader implications for intergenerational connections, well-being, and social cohesion.

